# Frontline therapies for untreated chronic lymphoid leukemia

**DOI:** 10.1186/s40164-019-0139-8

**Published:** 2019-08-17

**Authors:** Delong Liu, Juanjuan Zhao

**Affiliations:** 1 Division of Hematology & Oncology, New York Medical College, Valhalla, NY 10595 USA; 2 Department of Oncology, The First Affiliated Hospital of Zhengzhou University, Zhengzhou, 450052 China

**Keywords:** Chronic lymphoid leukemia, Venetoclax, Ibrutinib, ALLIANCE, iLLUMINATE, RESONATE-2

## Abstract

Therapy for chronic myeloid leukemia (CLL) is going through a major paradigm shift. Combination chemoimmunotherapy regimens have been the frontline therapies for CLL, whereas chlorambucil remained the standard frontline therapy for older patients (65 years or older) with CLL until recently. Monoclonal antibodies including rituximab, ofatumumab and obinutuzumab have been used for CLL therapy. Novel immunotherapeutics with chimeric antigen receptor (CAR) engineered T cells is rapidly migrating to clinical applications. Targeted therapies with small molecule inhibitors against Bruton tyrosine kinase (BTK) such as ibrutinib and acalabrutinib are playing a major role for treatment of patients with either treatment-naïve or refractory/relapsed CLL. Several major clinical trials including RESONATE-2, iLLUMINATE, ALLIANCE, ECOG 1912, CLL10, CLL14 as well as ibrutinib plus venetoclax have been ongoing in patients with untreated CLL. Frontline therapy of patients with untreated CLL appears to be shifting from chemotherapy to chemotherapy-free regimens. This review summarized latest development for frontline therapies of untreated CLL.

## Background

Therapy for chronic myeloid leukemia (CLL) is going through a major paradigm shift [1–3]. Combination chemoimmunotherapy regimens like FCR (fludarabine, cyclophosphamide, rituximab) and BR (bendamustine, rituximab) have been the frontline therapies for CLL, whereas chlorambucil remained the standard frontline therapy for older patients (65 years or older) with CLL until recently [4, 5]. Monoclonal antibodies including rituximab, ofatumumab and obinutuzumab have been used for CLL therapy [6, 7]. Novel immunotherapeutics with chimeric antigen receptor (CAR) engineered T cells is rapidly migrating to clinical applications [8–17]. Targeted therapy with small molecule inhibitors against Bruton tyrosine kinase (BTK) such as ibrutinib and acalabrutinib are playing a major role for treatment of patients with either treatment-naïve or refractory/relapsed CLL [18–22]. Several major clinical trials have been ongoing in patients with treatment-naïve CLL (TN CLL) [1]. Frontline therapy of patients with TN CLL appears to be shifting from chemotherapy to chemotherapy-free regimens. This review summarized latest development for frontline therapy of untreated CLL (Table 1).

**Table 1:** Clinical trials of frontline regimens for untreated chronic lymphoid leukemia

Study (references)	Regimens	Age (years)	del(17p) exclusion	No. patients	Follow-up month (range)	Response (RR; 95% CI; P)	PFS	OS	Undetectable MRD rate (sensitivity, 10^−4^)
RESONATE-2 [25]	Ibrutinib vs chlorambucil	≥ 65	Yes	269	18.4	ORR: 86% vs 35% (2.42; 1.91–3.07; P < 0.001) CR/CRi: 4% vs 2%	Median PFS not reached vs 18.9 months HR 0.16; 95% CI 0.09–0.28; P < 0.001 PFS rate at 18 months 90% vs 52%	Median OS not reached in either group OS rate at 24 months 98% vs 85% HR 0.16; 95% CI 0.05–0.56; P = 0.001	Not reported
ALLIANCE A041202 [26]	Ibrutinib vs IR vs BR	≥ 65	No	547	38	ORR: 93% vs 94% vs 81% CR: 7% vs 12% vs 26%	Median PFS not reported PFS rate at 24 months 87% vs 88% vs 74% HR 0.39; 0.26–0.58; P < 0.001 (BR vs I) HR 0.38; 0.25–0.59; P < 0.001 (BR vs IR) HR 1.00; 0.62–1.62; P = 0.49 (IR vs I)	Median OS not reported OS rate at 24 months 90% vs 94% vs 95% P ≥ 0.65 for all pairwise comparisons	In bone marrow: 1% vs 4% vs 8% (after 9 cycles)
ECOG E1912 [28]	IR vs FCR	18–70	Yes	529	33.4	Not reported	Median PFS HR 0.35; 0.22–0.5; P < 0.001 PFS rate not reported	Median OS HR 0.17; .05–0.54; P < 0.003 OS rate not reported	Not reported
iLLUMINATE [30]	IO vs CO	IO: 70 (66 to 75); CO: 72 (66–77)	No	229	31.3 (29.4–33.2)	ORR: 88% vs 73% (1.21; 1.06–1.37; P = 0.0035) CR/CRi: 22% vs 8% (2.51; 1.21–5.21; P = 0.0096)	Median PFS not reached vs 19.0 months (15.1–22.1) HR 0.23; 0.15–0.37; P < 0.0001 PFS rate at 30 months 79% vs 31%	Median OS not reached in either group HR 0.92; 0.48–1.77 OS rate at 30 months 86% vs 85%	In peripheral blood: 20% vs 30% In bone marrow: 17% vs 20%
Ibrutinib plus venetoclax [31]	Ibrutinib plus venetoclax	65 (26–83)	No	80	14.8	CR/CRi: 88% (after 12 cycles)	Median PFS not reported PFS rate at 12 months 98%; 95% CI 94–100	Median OS not reported OS rate at 12 months 99%; 95% CI 96–100	In bone marrow: 61% (after 12 cycles)
CLL14 [32]	VO vs CO	72 (41–89)	No	432	28.1	ORR: 84.7% vs 71.3% (P < 0.001) CR: 49.5% vs 23.1% (P < 0.001)	Median PFS not reported PFS rate at 24 months 88.2% vs 64.1%	Median OS not reached in either group OS rate at 24 months 91.8% vs 93.3% HR 1.24; 0.64–2.40; P = 0.52	In peripheral blood: 75.5% vs 35.2%; P < 0.001 In bone marrow: 56.9% vs 17.1%; P < 0.001 (3 months after treatment completion)
CLL10 [38]	BR vs FCR	FCR: 62.1 (55–67); BR: 61 (54–69)	Yes	561	37.1 (31.0–45.5)	ORR: 96% vs 95% (P = 1.0) CR: 31% vs 40% (P = 0.034)	Median PFS 41.7 months vs 55.2 months HR 1.643; 90.4% CI 1.308–2.064	Median OS not reported OS rate at 36 months 92% vs 91% HR 1.034; 0.620–1.724; P = 0.89	In peripheral blood: 38% vs 49%; P = 0.041 In bone marrow: 11% vs 27%; P < 0.001

*I* ibrutinib, *BR* bendamustine rituximab, *IR* ibrutinib rituximab, *VO* venetoclax obinutuzumab, *CO* chlorambucil obinutuzumab, *FCR* fludarabine cyclophosphamide rituximab, *CR* complete remission, *OS* overall survival, *PFS* progression free survival, *ORR* overall response rate, *MRD* minimal residual disease

## RESONATE-2 trial: ibrutinib vs chlorambucil

Traditionally chlorambucil was the standard agent for frontline therapy of elderly patients (> 65) with CLL [23, 24]. Ibrutinib was compared with chlorambucil in a phase 3 randomized multicenter international study, RESONATE-2, in untreated older patients (≥ 65 years) with CLL/SLL [25]. Patients with chromosome 17p13.1 deletion were excluded in this trial. PFS (progression free survival) was the primary end point. 269 patients with a median age of 73 were enrolled. Among these patients, 136 received ibrutinib (420 mg daily), 133 received chlorambucil. The median follow-up was 18.4 months. Ibrutinib led to a significant increase in PFS over chlorambucil (median, not reached vs. 18.9 months), with a hazard ratio of 0.16, P < 0.001. What is more striking is that ibrutinib as a single oral agent significantly prolonged OS. The relative risk of death for patients in the ibrutinib group was 84% lower than that in the chlorambucil group (hazard ratio, 0.16; P = 0.001). Ibrutinib was also found to have significantly higher ORR than chlorambucil (86% vs. 35%, P < 0.001). Severe hemorrhage was reported in 5 patients who received ibrutinib. Atrial fibrillation was observed in 6% of the patients who were taking ibrutinib over the period of 1.5 years. Hypertension was also found to be more frequent than those in the chlorambucil group. Therefore, in previously untreated older patients with CLL/SLL, ibrutinib was confirmed to be significantly better than chlorambucil in OS, PFS and ORR. The RESONATE-2 study for the first time placed ibrutinib as the standard frontline oral agent for this population of patients with CLL/SLL.

## ALLIANCE A041202 trial: ibrutinib vs ibrutinib/rituximab (IR) vs bendamustine/rituximab (BR)

Ibrutinib as a single agent was compared with bendamustine plus rituximab (BR) and ibrutinib plus rituximab (IR) in patients (≥ 65 years) with untreated CLL/SLL in a phase 3, randomized study, the ALLIANCE A041202 trial [26]. PFS was the primary end point. A total of 547 patients were enrolled, including 182 in the ibrutinib group, 182 in IR group, and 183 in the BR group. Median PFS was still not reached for the ibrutinib and IR groups at the time of this publication. The PFS at 2 years for the groups were 74% BR, 87% ibrutinib, and 88% IR. Compared with BR, the risk of death or disease progression was reduced by 61% in the ibrutinib group (HR = 0.39; 95% confidence interval [CI] 0.26 to 0.58; P < 0.001), and by 62% in the IR group (HR = 0.38; 95% CI 0.25 to 0.59; P < 0.001). PFS remained similar between ibrutinib and IR groups. Therefore, for patients with CLL and age 65 or older, continuous ibrutinib as well as IR was shown to be superior to six cycles of BR as assessed by PFS, though OS were similar among the three groups. It was postulated from in vitro studies that ibrutinib suppresses antibody-dependent cellular cytotoxicity, thereby rendering rituximab ineffective when the two were combined. This may explain in part that ibrutinib and IR had similar PFS. It is important to point out that at the time the study was designed, patients with chromosome 17p deletion were not excluded in this trial. It is clear now that these patients are inappropriate for BR therapy (n = 14 in the BR group), though patients who progressed in the BR group were allowed to cross over to receive ibrutinib. Atrial fibrillation of grade 3 and 4 was reported to be 3% in BR group, 9% in ibrutinib group, and 6% in IR group. The ALLIANCE study again independently confirmed that ibrutinib as a single agent is superior to BR combination regimen in this group of untreated older CLL patients in PFS.

## ECOG E1912 trial: ibrutinib/rituximab vs FCR (fludarabine, cyclophosphamide, rituximab)

FCR remains as the most active regimen in CLL/SLL patients younger than 70 years of age [27]. However, the median age of CLL/SLL patients are over 65. In addition, ibrutinib as a single agent is usually given continuously until disease progression or development of significant toxicity. Therefore, patients with CLL/SLL between age 18–70 were randomized to receive IR (ibrutinib/rituximab) or FCR in a 2:1 ratio [28]. Patients with chromosome 17p13.1 deletion were excluded in this trial. The primary end point was PFS, OS was the secondary end point. 529 patients were enrolled, with 354 patients in the IR group, and 175 in the FCR group. The median follow-up was 33.4 months. IR led to a 65% reduction in the risk of disease progression or death in comparison with FCR (HR = 0.35; 95% CI 0.22–0.5; P < 0.001). IR also significantly prolonged OS, with HR = 0.17 (95% CI 0.05–0.54; P < 0.003). Furthermore, IR regimen is significantly less toxic than FCR, since severe TEAE (treatment-emerging adverse event) in FCR group (72%) was significantly more frequent than that in the IR group (58%) (P = 0.0042).

In the subgroup analysis, patients with unmuted IGHV benefited more from IR than from FCR, with reduction of risk of death or disease progression by 74 percent by IR (HR = 0.26; 95% CI 0.14–0.5; P < 0.0001).

Therefore, in untreated patients with CLL/SLL, IR is superior to FCR in terms of PFS and OS as well as in therapy toxicity.

## iLLUMINATE trial: ibrutinib/obinutuzumab (IO) vs chlorambucil/obinutuzumab (CO)

Chlorambucil/obinutuzumab (CO) combination was shown to be superior to chlorambucil plus rituximab in CLL patient with comorbidities [29]. IO was compared to CO in an international, multicenter, randomized, open-label, phase 3 trial, iLLUMINATE, in untreated CLL/SLL patients ≥ 65, or younger patients with co-morbidities [30]. The primary end point was PFS. A total of 229 patients were enrolled, including 113 in IO group, and 116 in CO group. The median follow-up was 31.3 months (29.4–33.2). PFS in the IO group was significantly longer than that in CO group (HR = 0.23, 95% CI 0.15–0.37, P < 0.0001). Severe neutropenia and thrombocytopenia were the most common adverse events in both groups. Therefore, this chemotherapy-free combination regimen, IO, is a good option of frontline therapy in this group of older CLL/SLL patients or younger patient with co-morbidities. At this time, it is unclear whether IO is superior to ibrutinib alone. The rates of CR and undetectable minimal residual disease (MRD) appeared to favor IO combination over single agent ibrutinib, though the two have not been directly compared in a randomized trial.

## Ibrutinib plus venetoclax

Since ibrutinib and venetoclax have distinct mechanisms of action, the two may have synergistic activities. Ibrutinib and venetoclax as two oral agents were studied in a phase 2 trial in untreated CLL patients with one or more of the high risk features: chromosome 17p deletion, mutated TP53, chromosome 11q deletion, unmutated IGHV, 65 years or older [31]. To minimize the risk of tumor lysis, ibrutinib monotherapy at 420 mg daily were given for 3 cycles prior to combination with venetoclax (weekly dose escalation up to 400 mg once daily). The combination treatment was continued for 24 months. A total of 80 patients were enrolled, with a median age of 65 years (26–83 years). Responses to the combined treatment continued to improve over time and were noted across all subgroups with high-risk features. High CR/CRi were observed at 88% after 12 cycles of combined therapy, and 61% had deep remission with undetectable MRD which was assessed by means of multicolor flow cytometry in bone marrow (sensitivity, 10^−4^). Laboratory TLS was seen in three patients. This chemotherapy-free oral regimen appeared to be highly effective in the phase 2 trial for high-risk untreated CLL patients. Further randomized study is needed.

## CLL14 trial: venetoclax plus obinutuzumab (VenG) vs chlorambucil plus obinutuzumab (CO)

Venetoclax plus obinutuzumab (VenG) was compared with CO in previously untreated patients with CLL and comorbidities [32]. The treatment was given for a total of 12 cycles, with obinutuzumab for the first 6 cycles only. The primary end point was PFS, and the secondary end point was MRD negativity in the peripheral blood (PB) or bone marrow (BM) 3 months after completion of therapy. MRD was assessed every 3 months starting from cycle 4 by PCR and by next generation sequencing. A total of 432 patients were enrolled, with 216 in each arm. The median follow-up was 29 months. VenG was superior to CO in PFS (HR 0.35; 95% CI 0.23–0.53; P < 0.0001) as well as in MRD negativity in both PB (76% vs 35% [P < 0.0001]) and BM (57% vs 17% [P < 0.0001]). MRD negativity correlated with longer PFS. This correlation has been suggested in several analysis [33–36]. VenG induced higher and more sustainable MRD negativity. Therefore, this regimen represents the only fixed-duration chemotherapy-free treatment for untreated CLL with comorbidities [37]. Based on this CLL14 trial, this VenG regimen is now approved by FDA for first-line therapy of CLL/SLL.

## CLL10: BR vs FCR

Both BR and FCR have been used as frontline therapy for CLL/SLL, with FCR approved for younger patients whereas BR appears to have less toxicity. BR and FCR were compared in a randomized study, CLL10 [38]. Patients with 17p deletion were excluded. The primary end point was PFS. CLL10 was a non-inferiority trial. The intention-to-treat analysis was done in 561 eligible patients, including 282 patients in the FCR group and 279 in the BR group. The median follow-up was 37.1 months (range 31.0–45.5). The PFS for BR group was 41.7 months (95% CI 34.9–45.3) and 55.2 months (95% CI not reached) for FCR group (HR 1.643, 90.4% CI 1.308–2.064). Severe neutropenia and infection were more common in the FCR group. It appeared that in this group of clinically fit patients with untreated CLL, FCR is superior to BR and remains as the standard chemotherapy regimen, though BR is associated with less toxicity.

## Choice of a frontline regimen and special considerations

For treatment-naïve CLL patients with p53 mutation or 17p deletion, ibrutinib or chemotherapy-free regimen with venetoclax plus obinutuzumab (VenG) remains the preferred choice of therapy. For those untreated patients without p53 mutation or 17p deletion, based on data from the studies discussed above, NCCN guidelines (nccn.org) suggested ibrutinib as the preferred regimen. For frail patients with significant comorbidities or patients > 65 years of age, chemotherapy-free regimen with VenG can be considered as a preferred alternative. A variety of alternative regimens are available for considerations.

Additional clinical situations may warrant further careful considerations for making a reasonable choice of frontline regimen. For example, for a patient who is on anticoagulation for atrial fibrillation and/or venous thrombosis, it may be prudent to consider VenG instead of ibrutinib so that risk of bleeding can be minimized. Patients frequently ask how long the treatment will last at the initial discussion of therapy options. Currently ibrutinib therapy does not have a clear timeline for treatment termination. Therefore, if a patient wishes to only take a limited time of therapy, VenG is the only regimen to have a fixed 12-cycle duration.

## Conclusion

Small molecule BTK inhibitor, ibrutinib, is playing a central role in frontline therapy of untreated CLL since several major trials as outlined above provided evidence that ibrutinib as a single agent can be considered as the frontline therapy. Recently venetoclax plus obinutuzumab regimen has been approved as the first chemotherapy-free frontline therapy for untreated CLL with a fixed duration. Additional agents such as acalabrutinib are also under clinical trials for frontline therapy of untreated CLL [20, 39, 40].

## Data Availability

The material supporting the conclusion of this review has been included within the article.

## Abbreviations

I: ibrutinib
BR: bendamustine rituximab
IR: ibrutinib rituximab
VO: venetoclax obinutuzumab
CO: chlorambucil obinutuzumab
FCR: fludarabine cyclophosphamide rituximab
CR: complete remission
OS: overall survival
PFS: progression free survival
ORR: overall response rate
MRD: minimal residual disease
